# Designing Sandwich ELISA with Broadly Reactive Anti-Nucleocapsid Monoclonal Antibodies to Detect Bat-Borne Merbecoviruses

**DOI:** 10.3390/v17070886

**Published:** 2025-06-24

**Authors:** Kong Yen Liew, Yaju Wang, Sneha Sree Mullapudi, Dinah binte Aziz, Wenjie Fan, Min Luo, Paul Anantharajah Tambyah, Yee-Joo Tan

**Affiliations:** 1 Infectious Diseases Translational Research Programme and Department of Microbiology and Immunology, Yong Loo Lin School of Medicine, National University of Singapore, Singapore 117545, Singapore; kongyen@nus.edu.sg (K.Y.L.);; 2 Infectious Diseases Translational Research Programme and Department of Medicine, Yong Loo Lin School of Medicine, National University of Singapore, Singapore 117545, Singapore; 3 Department of Biological Sciences, Faculty of Science, National University of Singapore, Singapore 117558, Singapore

**Keywords:** betacoronavirus, nucleocapsid protein, monoclonal antibody, sandwich ELISA, bat-borne viruses, zoonotic surveillance

## Abstract

At least three betacoronaviruses have spilled over from bats to humans and caused severe diseases, highlighting the threat of zoonotic transmission. Thus, it is important to enhance surveillance capabilities by developing tools capable of detecting a broad spectrum of bat-borne betacoronaviruses. Three monoclonal antibodies (mAbs) targeting the nucleocapsid (N) protein were generated using recombinant N proteins from severe acute respiratory syndrome coronavirus 2 (SARS-CoV-2) and Middle East Respiratory Syndrome Coronavirus (MERS-CoV). The cross-reactivities of these mAbs were evaluated against a panel of betacoronaviruses. Sandwich ELISAs (sELISAs) were subsequently developed to detect bat-borne betacoronaviruses that have high zoonotic potential. Among the mAbs, 7A7 demonstrated the broadest cross-reactivity, recognizing betacoronaviruses from the *Sarbecovirus*, *Merbecovirus* and *Hibecovirus* subgenera. The first sELISA, based on mAbs 7A7 and 6G10, successfully detected N protein in all clinical swab samples from COVID-19 patients with cycle threshold (Ct) values < 25, achieving 75% positivity overall (12/16). Using this as a reference, a second sELISA was established by pairing mAb 7A7 with mAb 8E2, which binds to multiple merbecoviruses. This assay detected the N protein of two merbecoviruses, namely the human MERS-CoV and bat-borne HKU5-CoV, at high sensitivity and has a limit of detection (LOD) that is comparable to the first sELISA used successfully to detect COVID-19 infection. These broadly reactive mAbs could be further developed into rapid antigen detection kits for surveillance in high-risk populations with close contact with wild bats to facilitate the early detection of potential zoonotic spillover events.

## 1. Introduction

Coronavirus disease 2019 (COVID-19), caused by the severe acute respiratory syndrome coronavirus 2 (SARS-CoV-2), emerged as a global pandemic, leading to widespread social, health and economic disruption [1]. Prior to this, two other coronaviruses (CoVs) of bat origin, SARS-CoV and Middle East respiratory syndrome coronavirus (MERS-CoV), had crossed the species barrier to infect humans [2,3]. Another four human CoVs (HCoVs) are OC43-CoV, HKU1-CoV, NL63-CoV and 229E-CoV [4]. CoVs are divided into four genera, namely alpha, beta, gamma and deltacoronavirus. Among these, only alpha and betacoronaviruses are known to infect humans, and many of them are found in bats. In addition, whole genome sequencing revealed 96% similarity between SARS-CoV-2 and the bat-borne RaTG13-CoV, supporting the hypothesis that bats are the reservoir for SARS-CoV-2 [5,6].

Betacoronaviruses are classified into five different subgenera, namely *Embecovirus*, *Sarbecovirus*, *Merbecovirus*, *Nobecovirus* and *Hibecovirus*. While SARS-CoV and SARS-CoV-2 belong to the subgenus *Sarbecovirus*, MERS-CoV is classified under the subgenus *Merbecovirus* [7]. In addition, there are MERS-like CoVs like *Tylonycteris* bat coronavirus HKU4 (Ty-HKU4-CoV) and *Pipistrellus* bat coronavirus HKU5 (Pi-HKU5-CoV) [8]. HKU4-CoV utilizes dipeptidyl peptidase-4 (DPP4) as its entry receptor—the same receptor exploited by MERS-CoV. However, HKU4-CoV binds preferentially to bat DDP4, while MERS-CoV has adapted to bind to human DDP4 [9]. In contrast, HKU5-CoV and several other bat CoVs use bat angiotensin-converting enzyme 2 (ACE2) as their receptor [10,11]. In addition, HKU5-CoV-2 has been shown to use human ACE2 as a functional receptor [12], raising concern that HKU5-related viruses may have the potential to spill over into the human population [13].

Antigen detection assays detecting multiple strains of betacoronaviruses could be useful for monitoring spillover events. Diagnostic for COVID-19 has focused on detecting the nucleocapsid (N) protein of SARS-CoV-2 [14]. N is a structural protein that plays essential roles in viral replication and genome packaging [15]. It is highly abundant in the blood and saliva of COVID-19 patients [16,17]. However, existing COVID-19 Antigen Rapid Test (ART) kits are specific to SARS-CoV-2 and may not detect bat CoVs. To address this gap, broadly reactive anti-N monoclonal antibodies (mAbs) were generated from immunized mice. The binding of these mAbs to multiple bat-borne betacoronaviruses was evaluated, and they were formatted into sandwich enzyme-linked immunosorbent assay (ELISA) format to assess their sensitivity in detecting SARS-CoV-2 in COVID-19 clinical samples, as well as recombinant N proteins of MERS-CoV and HKU5-CoV.

## 2. Methods

### 2.1. Cell Lines, Virus and Protein Accession Numbers

293T/17, COS-7 and Vero E6 cells were purchased from the American Type Culture Collection (ATCC, Manassas, VA, USA) and cultured in Dulbecco’s Modified Eagle’s Medium (Hyclone, Logan, UT, USA) supplemented with 10% fetal bovine serum (FBS) (Hyclone). SARS-CoV-2 Omicron variant EG.5.1.1 (hCoV-19/Singapore/NUS0001/2023, GISAID accession: EPI_ISL_19016298) was propagated in Vero E6 cells, and viral titer was determined using plaque assay. The accession numbers of all N proteins are listed in Supplementary Table S1.

### 2.2. Recombinant N Proteins and mAbs

Recombinant HKU5-CoV N was purchased (Cusabio, Houston, TX, USA). SARS-CoV-2 N and MERS-CoV N were cloned into the pGEX-6P1 vector (GE Healthcare, Chicago, IL, USA), while ZJ-CoV N was cloned into the pET-22b vector. Bacterially expressed SARS-CoV-2 N and MERS-CoV N were purified using glutathione-Sepharose beads (GE Healthcare), while ZJ-CoV N was purified using IMAC. Mice were sequentially immunized with SARS-CoV-2 and MERS-CoV N proteins to produce the hybridomas, as described previously [18]. Animal experiments were performed according to the approved institutional animal care and use committee (IACUC) protocol (R22-0062) at the National University of Singapore. MAbs were purified from cell culture supernatants using protein G columns (GE Healthcare), and Coomassie Plus protein assay (Thermo Fisher Scientific, Waltham, MA, USA) was used to determine their concentrations.

### 2.3. Transient Transfection

All the HCoV N genes except OC43-CoV were generated previously [19]. The N gene was obtained by extracting viral RNA from OC43-CoV (ATCC), followed by reverse transcription-PCR and cloning into pXJ40-FLAG vector. The N genes of bat CoVs, RaTG13-CoV, BM48-CoV, HKU4-CoV, HKU5-CoV, HKU25-CoV, HKU5-CoV-2, SC2013-CoV, HKU9-CoV and CD35-CoV were codon-optimized, generated by gene synthesis (Bio Basic Asia Pacific or Genscript, Singapore) and subcloned into flag-tagged mammalian expression vector. For transient transfection, 293T cells were transfected using Lipofectamine 2000 reagent (Invitrogen, Carlsbad, CA, USA). At 24 h post-transfection, cells were harvested and lysed in RIPA buffer.

### 2.4. Western Blot (WB) and Primary Antibodies

Cell lysates were subjected to WB using rabbit anti-flag antibody (Millipore, Burlington, MA, USA) or home-made anti-N mAbs as primary antibody. After the addition of HRP-conjugated secondary antibodies, the membranes were imaged with enhanced chemiluminescence substrate (Thermo Fisher Scientific) and ChemiDoc MP Imaging System (Bio-Rad, Hercules, CA, USA).

### 2.5. Sandwich ELISA (sELISA) and Limit of Detection (LOD)

Home-made mAb (0.2 µg/well) was diluted in coating buffer and coated onto 96-well plates overnight at 4 °C. Plates were washed with PBST (0.1% Tween 20 in PBS) and blocked with 5% FBS in PBST before incubation with diluted proteins in 1% BSA-PBST for 3 h at 37 °C. Alternatively, diluted SARS-CoV-2 virion was mixed with equal amounts of virion lysis buffer [50 mM Tris-HCl (pH 8), 150 mM NaCl and 1% Triton X-100] and incubated on ice for 1 h before being added to the plate. Next, biotin-labeled mAb was added and incubated for 1.5 h at 37 °C, followed by streptavidin-HRP (Thermo Fisher Scientific) for 1 h at 37 °C. TMB substrate was then added for 30 min, followed by 2M sulfuric acid and absorbance measurement. Biotin-labeling was performed using EZ-Link™ Sulfo NHS-LC-LC-Biotin and Zeba™ Spin Columns (Thermo Fisher Scientific).

For each sELISA, the cut-off value was determined as the mean value of OD450 nm derived from at least 6 blank wells plus three times their standard deviation (mean + 3xSD). The limit of detection (LOD) of each sELISA is determined as the lowest concentration of analyte that produces a signal higher than the cut-off.

### 2.6. Nasal Swabs from COVID-19 Patients

Nasal swabs were obtained from 16 COVID-19 patients hospitalized at the National University Hospital, Singapore. The patients were recruited under the NOFA study (Ethics and Compliance Online System, ECOS, ref. 2023/00365) and provided written informed consent for participation in the study. Ethical approval for the NOFA study was obtained from the ethics committee of Singapore’s National Health Group Domain Specific Review Board, now known as ECOS. Patients were swabbed by a trained study team member using BD™ Universal Viral Transport System (Becton Dickinson and Company, Franklin Lakes, NJ, USA). After vortexing, the sample was aliquoted into sterile screw cap tubes and stored at −80 °C until further use. The COVID-19 patients were recruited over a period of ~7 months, and then, the samples were thawed and tested at the same time. Thus, all the samples were used with 1 freeze–thaw cycle and within 1 year of freezing. Swab samples were tested in sELISA in the same manner as above, except that the swab sample was combined with a one-tenth volume of 10x stock of virion lysis buffer.

### 2.7. Quantification of SARS-CoV-2 Viral Ribonucleic Acid (RNA) Level

Viral RNA was isolated from 140 μL of virus suspension using QIAamp Viral RNA Mini Kit (Qiagen, Venlo, The Netherlands) with an elution volume of 60 μL. One-step qRT-PCR was performed using 5 μL of the extracted viral RNA for each 25 μL reaction according to the recommended cycling conditions of SuperScript™ III Platinum™ SYBR™ Green One-Step qRT-PCR Kit (Invitrogen). The primers used to detect SARS-CoV-2 N gene (CCDC-N-F: 5′-GGG GAA CTT CTC CTG CTA GAA T-3′ and CCDC-N-R: 5′-CAG ACA TTT TGC TCT CAA GCT G-3′) were previously published [20].

### 2.8. Statistical Analysis

A two-tailed unpaired Student’s *t*-test was performed to assess the statistical significance between datasets derived from three independent experiments. A *p*-value < 0.05 was considered statistically significant.

## 3. Results

### 3.1. Identification of a Broadly Reactive mAb 7A7 Binding to Highly Pathogenic Human Betacoronaviruses

Three antibodies, namely, mAb 6G10, mAb 8E2 and mAb 7A7, were generated via sequential immunization of mice with SARS-CoV-2 and MERS-CoV N proteins. Indirect ELISA was used to ascertain their preferential binding and EC_50_ values were computed to estimate their binding affinities to N proteins. As shown in Table 1, mAb 6G10 demonstrated no binding to MERS-CoV N but exhibited strong binding to SARS-CoV-2 N. In contrast, mAb 8E2 showed strong binding to MERS-CoV N. Among the three, mAb 7A7 binds both SARS-CoV-2 N and MERS-CoV N with low EC_50_ (Table 1) and detected N in SARS-CoV-2-infected Vero E6 (Supplementary Figure S1).

**Figure 1:**
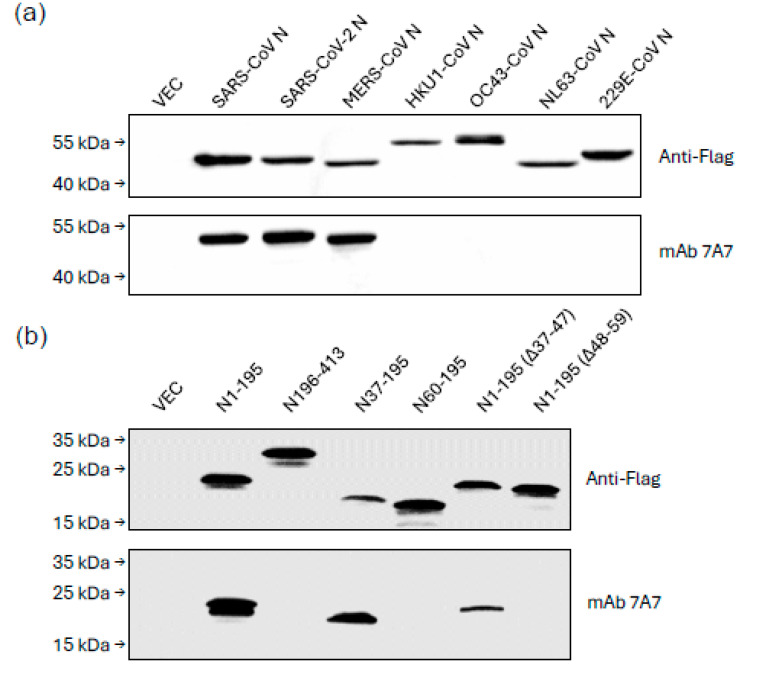
mAb 7A7 binds to highly pathogenic human betacoronaviruses. Lysates from 293T cells transiently transfected with Flag-tagged (**a**) N protein plasmids of beta and alpha coronaviruses; (**b**) truncated MERS-CoV N mutants were immunoblotted with anti-Flag Ab and mAb 7A7. Empty vector-transfected 293T cell lysate (VEC) serves as a negative control.

The breadth of mAb 7A7 binding was evaluated using WB of 293T cells transfected with flag-tagged N plasmids. As shown in Figure 1a, mAb 7A7 bound N of SARS-CoV, SARS-CoV-2 and MERS-CoV but not of endemic betacoronaviruses (HKU1-CoV, OC43-CoV) and alphacoronaviruses (NL63-CoV and 229E-CoV). To identify the epitope of mAb 7A7, MERS-CoV-N truncation mutants were used. MAb 7A7 bound to N1-195 and N37-195 but not to N60-195, suggesting that the antibody’s binding epitope is within the 37–59 amino acids (AA) of N1-195 (Figure 1b). Furthermore, mAb 7A7 did not bind to N1-195(Δ48–59), confirming that its binding epitope lies in 48–59 AA (^48^TQHGKVPLTFPP^59^) of N1-195.

### 3.2. Binding of mAb 7A7 to Bat CoVs from the Sarbecovirus, Merbecovirus and Hibecovirus Subgenera

**Figure 2:**
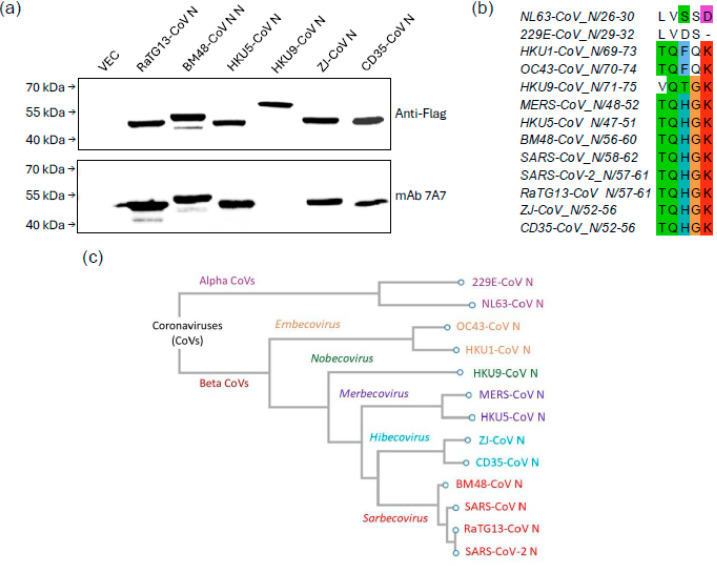
mAb 7A7 binds to *Sarbecovirus*, *Merbecovirus* and *Hibecovirus*. (**a**) Lysates from 293T cells transiently transfected with Flag-tagged N protein plasmids of 6 different bat CoVs were immunoblotted with anti-Flag Ab and mAb 7A7. (**b**) Sequence alignment of N proteins highlights the conserved binding epitope ‘TQHGK’ targeted by mAb 7A7. (**c**) Phylogenetic tree based on N protein sequences of alpha and betacoronaviruses.

MAb 7A7 was tested against bat CoVs from different subgenera, including *Sarbecovirus*, *Merbecovirus*, *Nobecovirus* and *Hibecovirus*. WB (Figure 2a) and immunofluorescence assay (Supplementary Figure S1) revealed that mAb 7A7 bound to the N proteins of bat-borne Sarbecoviruses (RaTG13-CoV and BM48-CoV), Merbecovirus (HKU5-CoV) and Hibecovirus (ZJ-CoV and CD35-CoV) but not to that of Nobecovirus (HKU9-CoV). Sequence alignment shows that the epitope ‘TQHGK’ is present in betacoronaviruses bound by mAb 7A7 and absent in those it did not recognize (Figure 2b). This epitope, located in the 48–52 AA region of N1-195, aligns with the binding region identified through WB using truncated MERS-CoV N mutants (Figure 1b). Furthermore, phylogenetic analysis based on N protein sequences also showed that betacoronaviruses from *Sarbecovirus*, *Merbecovirus* and *Hibecovirus* are genetically close to one another (Figure 2c). In contrast, mAb 7A7′s epitope is not conserved in the evolutionarily distant Embecovirus and Nobecovirus.

### 3.3. First sELISA for Detecting SARS-CoV-2 Virions and COVID-19 Patients

**Figure 3:**
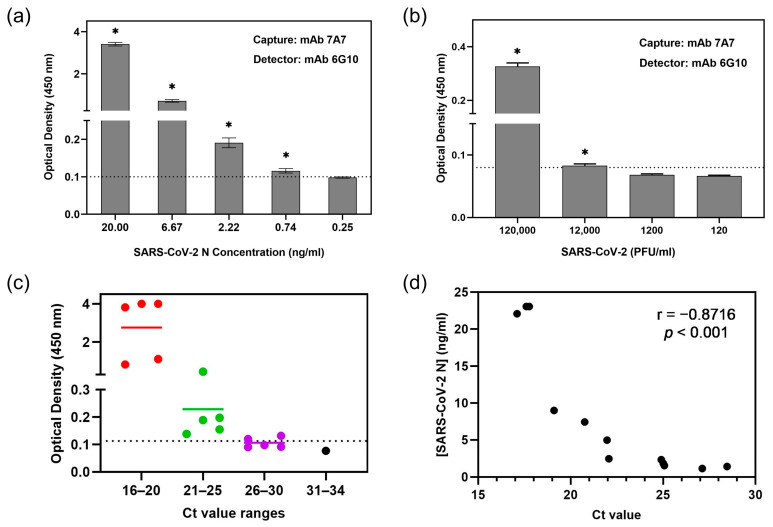
sELISA using mAb 7A7(6G10) detects SARS-CoV-2. mAb 7A7 and biotinylated-mAb 6G10 were used as capture and detector antibodies, respectively, in sELISA of SARS-CoV-2 (**a**) N protein and (**b**) virions. * *p* < 0.05 represents significant difference compared to blank. (**c**) Plot of individual OD_450nm_ readings of 16 COVID-19 patients’ nasal swabs in 7A7(6G10) sELISA, which are categorized into different Ct value ranges. Dotted line indicates the cut-off value. (**d**) Correlation between SARS-CoV-2 N protein concentrations and Ct values showing the Pearson correlation coefficient (r) value.

Another mAb 6G10 demonstrated strong binding to SARS-CoV-2 N protein with an EC_50_ of 0.43 μg/mL (Table 1) but did not bind to other human CoVs (Supplementary Figure S2). So the first sELISA was set up using mAb 7A7 as the capture antibody and biotinylated-6G10 as the detector antibody (denoted as 7A7(6G10)). As illustrated in Figure 3a, this sELISA detected SARS-CoV-2 N protein with a limit of detection (LOD) of 0.74 ng/mL. It also detected SARS-CoV-2 virions, with the lowest detectable amount being 12,000 PFU/mL (Figure 3b), corresponding to a Ct value of around 27 (Supplementary Table S2).

To determine whether this sELISA is suitable for detecting SARS-CoV-2 in patient samples, it was used to measure N in nasal swabs from 16 COVID-19 patients. RNA was also extracted from the same samples for qRT-PCR. Twelve out of the sixteen samples (75%) tested positive by sELISA (sample OD_450nm_ > cut-off OD_450nm_), and qRT-PCR showed that these have Ct values ≤ 28 (Figure 3c). Among the four samples that were negative in sELISA, three of them have Ct values of 26–30, while one has a Ct value of 33.5 (Supplementary Table S3). N protein concentrations in the swab samples were computed using an internal standard curve (Supplementary Figure S3). As expected, an inverse correlation was observed between Ct values and N protein concentration (Pearson correlation coefficient r = −0.8716, *p* < 0.001) (Figure 3d).

### 3.4. Second sELISA for Detecting MERS-CoV and Bat-Borne Merbecoviruses

**Figure 4:**
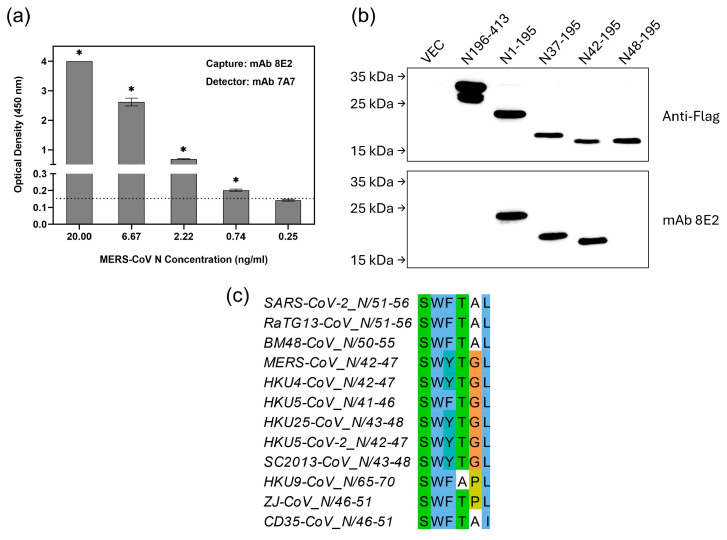
sELISA using mAb 8E2(7A7) detects MERS-CoV. (**a**) mAb 8E2 and biotinylated-mAb 7A7 were used as capture and detector antibody, respectively, in sELISA of MERS-CoV N protein. * *p* < 0.05 represents significant difference compared to blank. Dotted line indicates the cut-off value. (**b**) Lysates from 293T cells transiently transfected with Flag-tagged truncated MERS-CoV N mutants were immunoblotted with anti-Flag Ab and mAb 8E2. (**c**) Sequence alignment of N proteins highlights the potential region targeted by mAb 8E2.

MERS-CoV is another betacoronavirus with ancestral origins in bats that has spilled over into humans [2]. Since another mAb 8E2 bound strongly to MERS-CoV N protein (Table 1), it was used to set up a second sELISA. As shown in Figure 4a, the sELISA using 8E2(7A7), i.e., 8E2 as capture antibody and biotinylated 7A7 as detector antibody, achieved an LOD of 0.74 ng/mL. Given that MERS-CoV infections are rare and geographically restricted, we were unable to run sELISA with patient samples. Nevertheless, this LOD is comparable to that of the first sELISA (based on 7A7(6G10)) described above for detecting SARS-CoV-2 in COVID-19 patients.

WB using truncated MERS-CoV N proteins shows that residues 42 to 47 are essential for 8E2 binding (Figure 4b). In addition to MERS-CoV, multiple merbecoviruses have been identified in bats across various countries [21,22]. Sequence alignment shows that the epitope (^42^SWYTG^47^) recognized by 8E2 is highly conserved among bat-borne merbecoviruses but shows notable variation in the N proteins of bat CoVs from the *Sarbecovirus* (BM48-CoV and RaTG13-CoV), *Nobecovirus* (HKU9-CoV) and *Hibecovirus* (ZJ-CoV and CD35-CoV) subgenera (Figure 4c).

**Figure 5:**
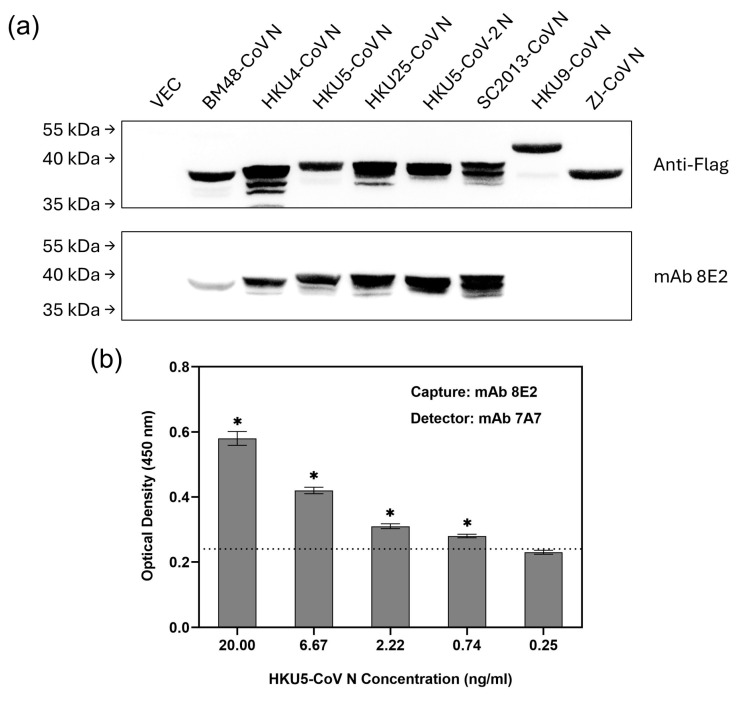
sELISA with mAb 8E2(7A7) detects bat-borne merbecoviruses. (**a**) Lysates from 293T cells transiently transfected with Flag-tagged N protein plasmids of 8 different bat CoVs were immunoblotted with anti-Flag Ab and mAb 7A7. (**b**) mAb 8E2 and biotinylated-mAb 7A7 were used as capture and detector antibody, respectively, in sELISA of HKU5-CoV N protein. * *p* < 0.05 represents significant difference compared to blank. Dotted line indicates the cut-off value.

Consistent with the conservation of its epitope, mAb 8E2 bound strongly to the N proteins of several bat-borne merbecoviruses, including HKU4-CoV, HKU5-CoV, HKU25-CoV, HKU5-CoV-2 and SC2013-CoV (Figure 5a). In contrast, it showed weaker binding to the N protein of BM48-CoV and did not bind to the N proteins of HKU9-CoV or ZJ-CoV. Although HKU5-CoV N has a phenylalanine (F) at position 44 instead of the tyrosine (Y) found in MERS-CoV N, mAb 8E2 still bound strongly to it, suggesting that residue Y44 is not critical for binding. Since both mAbs 7A7 and 8E2 bind to HKU5-CoV N in indirect ELISA (Table 1), the second ELISA based on the 8E2(7A7) pairing was tested using recombinant HKU5-CoV N protein. It successfully detected HKU5-CoV N with an LOD of 0.74 ng/mL (Figure 5b), the same as observed for MERS-CoV N (Figure 4a). In addition to HKU5-CoV, this second ELISA should also be suitable for detecting other bat-borne merbecoviruses, namely HKU4-CoV, HKU25-CoV, HKU5-CoV-2 and SC2013-CoV, as WB shows that both mAbs 7A7 and 8E2 can bind to their N proteins (Figure 5a and Supplementary Figure S4a). 7A7′s epitope ‘TQHGK’ is also conserved among these bat-borne merbecoviruses (Supplementary Figure S4b).

## 4. Discussion

Besides molecular detection of viral RNA, antigen detection assays are also commonly used to confirm an infection, especially in surveillance studies. In the case of SARS-CoV-2, the detection of the N protein by mAb led to the development of rapid antigen tests, which serve as point-of-care diagnostic devices. While these tests are less sensitive than molecular methods, they are cheaper and play a key role in the management of the COVID-19 pandemic [23]. While many antigen detection tests are available for SARS-CoV-2, there are few studies describing broadly reactive mAbs that can bind to the N proteins of multiple bat CoVs.

In this study, mice were sequentially immunized with recombinant N proteins of SARS-CoV-2 and MERS-CoV to generate mAbs with broad reactivities to betacoronaviruses from the *Sarbecovirus* and *Merbecovirus* subgenera. Among the generated antibodies, mAb 7A7 exhibited strong binding to the N proteins of SARS-CoV-2 and MERS-CoV, as well as broad cross-reactivity with N proteins of multiple betacoronaviruses from the subgenera of *Sabecovirus*, *Merbecovirus* and *Hibecovirus*, but not *Nobecovirus* (HKU9-CoV). The binding epitope of mAb 7A7 was identified as ‘TQHGK’, which is conserved among all tested betacoronaviruses except Embecovirus and Nobecovirus.

To evaluate the diagnostic potential of mAb 7A7, it was paired with mAb 6G10 to form a sandwich ELISA (sELISA). WB revealed that mAb 6G10 likely recognizes a distinct epitope from mAb 7A7 (Figure 1a and Supplementary Figure S2), making them a suitable pair. This 7A7(6G10) sELISA pairing effectively detected recombinant SARS-CoV-2 N protein at an LOD of 0.74 ng/mL (Figure 3a), which is comparable to other reported assays (LOD: 0.78–0.93 ng/mL) [24,25]. Furthermore, this sELISA detected SARS-CoV-2 virions at 12,000 PFU/mL, corresponding to a Ct value of 27 in qRT-PCR (Figure 3b and Supplementary Table S2). When tested on nasal swabs from COVID-19 patients, the assay detected N protein in 12 out of 16 PCR-positive samples (75%). Notably, all samples with Ct < 25 (n = 10) were positive, whereas only 2 of 5 samples with Ct values between 26 and 30 had detectable N protein. Thus, the sensitivity of this sELISA is similar to several commercial antigen-based rapid diagnostic tests, which detect moderately high percentages of SARS-CoV-2 samples within the Ct range of 25 to 30 [26].

In addition to SARS-like coronaviruses, MERS-like coronaviruses pose significant zoonotic risks. For instance, co-infections of MERS-CoV and bat HKU8-related coronaviruses were found in Kenyan camels [27]. Furthermore, pangolin-derived MERS-like coronavirus (MjHKU4r-CoV-1), which is closely related to BatCoV HKU4, was shown to infect human tissues and transgenic mice [28]. Therefore, a second sELISA was set up by pairing mAb 7A7 with mAb 8E2, a MERS-CoV N-specific antibody (Table 1). This combination successfully detected recombinant MERS-CoV N protein at a concentration as low as 0.74 ng/mL (Figure 4a). Given that human infections by MERS-CoV have only been reported in a few countries, testing of nasal swabs was not performed due to limited access to clinical samples. However, since our second sELISA has a similar LOD for recombinant N protein as the first sELISA (7A7 and 6G10 pairing), which detected most of the COVID-19 patients tested, this second sELISA is likely suitable for detecting human MERS-CoV infections.

Importantly, the epitope of mAb 8E2 is highly conserved among bat-borne merbecoviruses (Figure 4c), and WB confirmed its ability to bind to the N proteins from multiple merbecoviruses (Figure 5a). This includes the bat CoV HKU5, which has high zoonotic potential, as a recent study reported HKU5-CoV detection in the lungs and intestines of minks associated with a pneumonia outbreak on a farm [29]. Consistent with the broad reactivities of mAbs 7A7 and 8E2 for the N proteins of bat-borne merbecoviruses, our second sELISA using this pairing could detect recombinant HKU5-CoV N protein with high sensitivity (LOD of 0.74 ng/mL). Notably, the epitopes of mAbs 7A7 and 8E2 are also conserved in the recently discovered HKU5-CoV-2 (Figure 4c and Supplementary Figure S4b), which utilizes human ACE2 as its receptor [12]. sELISAs developed in this study using these broadly reactive anti-N mAbs also showed good reproducibility—the same LOD was achieved using different batches of mAbs 7A7 and 8E2 (Supplementary Figures S5 and S6)—suggesting that they could be useful for further applications.

## 5. Conclusions

In conclusion, we have developed and characterized a panel of anti-N mAbs with broad reactivity with multiple betacoronaviruses. Among them, mAb 7A7 exhibits the broadest cross-reactivity with betacoronaviruses from the *Sarbecovirus*, *Merbecovirus* and *Hibecovirus* subgenera. The first sELISA, based on mAb 7A7 and 6G10, detected SARS-CoV-2 virions and N protein in swab samples of COVID-19 patients. On the other hand, mAb 8E2 is another antibody with preferential binding to merbecoviruses. The second sELISA, using mAb 7A7 and 8E2, detected low concentrations of recombinant MERS-CoV and HKU5-CoV N proteins. Thus, these broadly reactive mAbs could be useful for developing diagnostic tools for surveillance of MERS-like viruses. Such test kits could possibly be deployed among wildlife market workers, miners, laboratory personnel or other high-risk populations to facilitate the early detection of potential zoonotic spillover.

## Figures and Tables

**Table 1:** EC50 of mAb 6G10, 8E2 and 7A7 measured by indirect ELISA against recombinant N proteins of SARS-CoV-2, MERS-CoV, HKU5-CoV and ZJ-CoV.

mAbs (Isotype)	EC50 (µg/mL)			
	SARS-CoV-2 N	MERS-CoV N	HKU5-CoV N	ZJ-CoV N
6G10 (IgG1) *	0.43 ± 0.04	n.d.	n.d.	n.d.
8E2 (IgG1)	19 ± 1.1	0.53 ± 0.08	2.7 ± 0.6	>20
7A7 (IgG1)	0.025 ± 0.004	0.004 ± 0.0006	0.02 ± 0.006	0.23 ± 0.04

* n.d.—not determined because WB shows that 6G10 is specific to SARS-CoV-2 (see Supplementary Figure S2).

## Data Availability

All the data supporting the findings of this study are available within the main manuscript and the Supplementary Materials. Further inquiries can be directed to the corresponding author.

## Supplementary Materials

The following supporting information can be downloaded at: https://www.mdpi.com/article/10.3390/v17070886/s1, Figure S1: Vero-E6 cells were mock-infected or infected with SARS-CoV-2 (multiplicity of infection of 0.1) and stained with mAb 7A7 at 2 days post-infection. (b) IFA results of Cos-7 cells expressing Flag-tagged vector or N proteins of HCoVs (top panel) or Flag-tagged N protein of BatCoVs (bottom panel) using mAb 7A7, followed by Alexa Fluor 488-conjugated secondary antibody (green) are shown. Cell nuclei were counterstained with DAPI (blue); Figure S2: Lysates from 293T cells transiently transfected with Flag-tagged N protein plasmids of 5 different betacoronaviruses belonging to the *Sarbecovirus* (SARS-CoV-2), *Merbecovirus* (MERS-CoV), *Nobecovirus* (HKU9-CoV) and *Hibecovirus* (ZJ-CoV and CD35-CoV) subgenera were obtained and immunoblotted with anti-Flag Ab and mAb 6G10. Empty vector-transfected 293T cell lysate labelled as ‘VEC’ served as a negative control. The molecular weight (kDa) of the proteins relative to their position on the blots is given on the left side of the panel; Figure S3: Standard curve between SARS-CoV-2 N protein concentrations and their respective OD_450nm_ from sELISA using 7A7(6G10). R^2^ value is calculated based on 4PL regression analysis; Figure S4: (a) Lysates from 293T cells transiently transfected with Flag-tagged N protein plasmids of 5 different merbecoviruses (HKU4-CoV, HKU5-CoV, HKU25-CoV, HKU5-CoV-2 and SC2013-CoV) were obtained and immunoblotted with anti-Flag Ab and mAb 7A7. The molecular weight (kDa) of the proteins relative to their position on the blots is given on the left side of the panel. (b) Sequence alignment of N proteins highlight the potential region targeted by mAb 7A7; Figure S5: sELISA using a different batch of mAb 7A7 to pair with mAb 6G10 achieves the same LOD for SARS-CoV-2 virions—12,000 PFU/mL (as shown in Figure 3b in the main text). mAb 7A7 and biotinylated-mAb 6G10 were used as capture and detector antibody, respectively, in sELISA of SARS-CoV-2 virions. * *p* < 0.05 represents significant difference compared to blank. Dotted line indicates the cut-off value; Figure S6: sELISA using a different batch of mAb 8E2 to pair with mAb 7A7 achieves the same LOD for recombinant HKU5-CoV N protein—0.74 ng/mL (as shown in Figure 5b in the main text). mAb 8E2 and biotinylated-mAb 7A7 were used as capture and detector antibody, respectively, in sELISA of HKU5-CoV N protein. * *p* < 0.05 represents significant difference compared to blank. Dotted line indicates the cut-off value; Table S1: Accession numbers of N proteins from different CoVs; Table S2: Ct values of SARS-CoV-2 virions with titres of 12,000 to 120 PFU/mL analysed in qRT-PCR; Table S3: N protein levels in the nasal swabs collected from 16 COVID-19 patients determined using sELISA and their Ct values obtained via qRT-PCR.

## References

[1] Nicola M., Alsafi Z., Sohrabi C., Kerwan A., Al-Jabir A., Iosifidis C., Agha M., Agha R. (2020). The socio-economic implications of the coronavirus pandemic (COVID-19): A review. Int. J. Surg..

[2] Memish Z.A., Mishra N., Olival K.J., Fagbo S.F., Kapoor V., Epstein J.H., Alhakeem R., Durosinloun A., Asmari M.A., Islam A. (2013). Middle East respiratory syndrome coronavirus in bats, Saudi Arabia. Emerg. Infect. Dis..

[3] Li W., Shi Z., Yu M., Ren W., Smith C., Epstein J.H., Wang H., Crameri G., Hu Z., Zhang H. (2005). Bats Are Natural Reservoirs of SARS-Like Coronaviruses. Science.

[4] Cui J., Li F., Shi Z.L. (2019). Origin and evolution of pathogenic coronaviruses. Nat. Rev. Microbiol..

[5] Zhou P., Yang X.-L., Wang X.-G., Hu B., Zhang L., Zhang W., Si H.-R., Zhu Y., Li B., Huang C.-L. (2020). A pneumonia outbreak associated with a new coronavirus of probable bat origin. Nature.

[6] Andersen K.G., Rambaut A., Lipkin W.I., Holmes E.C., Garry R.F. (2020). The proximal origin of SARS-CoV-2. Nat. Med..

[7] Llanes A., Restrepo C.M., Caballero Z., Rajeev S., Kennedy M.A., Lleonart R. (2020). *Betacoronavirus* Genomes: How Genomic Information has been Used to Deal with Past Outbreaks and the COVID-19 Pandemic. Int. J. Mol. Sci..

[8] Frutos R., Serra-Cobo J., Pinault L., Roig M.L., Devaux C.A. (2021). Emergence of Bat-Related Betacoronaviruses: Hazard and Risks. Front. Microbiol..

[9] Yang Y., Du L., Liu C., Wang L., Ma C., Tang J., Baric R.S., Jiang S., Li F., Yang Y. (2014). Receptor usage and cell entry of bat coronavirus HKU4 provide insight into bat-to-human transmission of MERS coronavirus. Proc. Natl. Acad. Sci. USA.

[10] Ma C.-B., Liu C., Park Y.-J., Tang J., Chen J., Xiong Q., Lee J., Stewart C., Asarnow D., Brown J. (2025). Multiple independent acquisitions of ACE2 usage in MERS-related coronaviruses. Cell.

[11] Park Y.-J., Liu C., Lee J., Brown J.T., Ma C.-B., Liu P., Gen R., Xiong Q., Zepeda S.K., Stewart C. (2025). Molecular basis of convergent evolution of ACE2 receptor utilization among HKU5 coronaviruses. Cell.

[12] Chen J., Zhang W., Li Y., Liu C., Dong T., Chen H., Wu C., Su J., Li B., Zhang W. (2025). Bat-infecting merbecovirus HKU5-CoV lineage 2 can use human ACE2 as a cell entry receptor. Cell.

[13] (2025). Newfound bat virus that uses notorious receptor poses ‘spillover’ risk. Nature.

[14] Diao B., Wen K., Zhang J., Chen J., Han C., Chen Y., Wang S., Deng G., Zhou H., Wu Y. (2021). Accuracy of a nucleocapsid protein antigen rapid test in the diagnosis of SARS-CoV-2 infection. Clin. Microbiol. Infect..

[15] Cubuk J., Alston J.J., Incicco J.J., Singh S., Stuchell-Brereton M.D., Ward M.D., Zimmerman M.I., Vithani N., Griffith D., Wagoner J.A. (2021). The SARS-CoV-2 nucleocapsid protein is dynamic, disordered, and phase separates with RNA. Nat. Commun..

[16] Bai Z., Cao Y., Liu W., Li J. (2021). The SARS-CoV-2 Nucleocapsid Protein and Its Role in Viral Structure, Biological Functions, and a Potential Target for Drug or Vaccine Mitigation. Viruses.

[17] Zhang Y., Ong C.M., Yun C., Mo W., Whitman J.D., Lynch K.L., Wu A.H.B. (2021). Diagnostic Value of Nucleocapsid Protein in Blood for SARS-CoV-2 Infection. Clin. Chem..

[18] Oh H.-L.J., Åkerström S., Shen S., Bereczky S., Karlberg H., Klingström J., Lal S.K., Mirazimi A., Tan Y.-J. (2010). An Antibody against a Novel and Conserved Epitope in the Hemagglutinin 1 Subunit Neutralizes Numerous H5N1 Influenza Viruses. J. Virol..

[19] Aboagye J.O., Yew C.W., Ng O.W., Monteil V.M., Mirazimi A., Tan Y.J. (2018). Overexpression of the nucleocapsid protein of Middle East respiratory syndrome coronavirus up-regulates CXCL10. Biosci. Rep..

[20] Kilic T., Weissleder R., Lee H. (2020). Molecular and Immunological Diagnostic Tests of COVID-19: Current Status and Challenges. iScience.

[21] Silvério B.S., Guilardi M.D., Martins J.O., Duro R.L.S., Sousa L.L.F.D., Cabral-Miranda G., Janini L.M.R., Poon L.L.M., Durães-Carvalho R. (2025). Coronavirus Cryptic Landscape and Draft Genome of a Novel CoV Clade Related to MERS From Bats Circulating in Northeastern Brazil. J. Med. Virol..

[22] Lau S.K.P., Li K.S.M., Tsang A.K.L., Lam C.S.F., Ahmed S., Chen H., Chan K.-H., Woo P.C.Y., Yuen K.-Y. (2013). Genetic Characterization of *Betacoronavirus* Lineage C Viruses in Bats Reveals Marked Sequence Divergence in the Spike Protein of *Pipistrellus* Bat Coronavirus HKU5 in Japanese Pipistrelle: Implications for the Origin of the Novel Middle East Respiratory Syndrome Coronavirus. J. Virol..

[23] Peeling R.W., Heymann D.L., Teo Y.-Y., Garcia P.J. (2022). Diagnostics for COVID-19: Moving from pandemic response to control. Lancet.

[24] Stokanic M.M., Simovic A., Jovanovic V., Radomirovic M., Udovicki B., Ristivojevic M.K., Djukic T., Vasovic T., Acimovic J., Sabljic L. (2023). Sandwich ELISA for the Quantification of Nucleocapsid Protein of SARS-CoV-2 Based on Polyclonal Antibodies from Two Different Species. Int. J. Mol. Sci..

[25] Lv H., Shi F., Yin H., Jiao Y., Wei P. (2024). Development of a double-antibody sandwich ELISA for detection of SARS-CoV-2 variants based on nucleocapsid protein-specific antibodies. Microbiol. Immunol..

[26] Patriquin G., LeBlanc J.J., Williams C., Hatchette T.F., Ross J., Barrett L., Davidson R. (2022). Comparison between Nasal and Nasopharyngeal Swabs for SARS-CoV-2 Rapid Antigen Detection in an Asymptomatic Population, and Direct Confirmation by RT-PCR from the Residual Buffer. Microbiol. Spectr..

[27] Zhang W., Zheng X.-S., Agwanda B., Ommeh S., Zhao K., Lichoti J., Wang N., Chen J., Li B., Yang X.-L. (2019). Serological evidence of MERS-CoV and HKU8-related CoV co-infection in Kenyan camels. Emerg. Microbes Infect..

[28] Zhao Z., Li X., Chai Y., Liu Z., Wang Q., Gao G.F. (2024). Molecular basis for receptor recognition and broad host tropism for merbecovirus MjHKU4r-CoV-1. EMBO Rep..

[29] Zhao J., Wan W., Yu K., Lemey P., Pettersson J.H., Bi Y., Lu M., Li X., Chen Z., Zheng M. (2024). Farmed fur animals harbour viruses with zoonotic spillover potential. Nature.

