# Supplementary material for: Designing Sandwich ELISA with Broadly Reactive Anti-Nucleocapsid Monoclonal Antibodies to Detect Bat-Borne Merbecoviruses
Source: Viruses. 2025 Jun 24;17(7):886. doi: 10.3390/v17070886 (PMC12300527; doi:10.3390/v17070886)
Supplement: Supplementary file 1 [file viruses-17-00886-s001.zip › viruses-3674842-supplementary.pdf]

Supplementary Material

Materials and Methods

Indirect enzyme-linked immunosorbent assay (ELISA)

Recombinant proteins (50 ng/well) were diluted in coating buffer (0.1M $NaHCO_3$, 34mM Na_2CO_3) and coated onto 96-well plates (Nunc) overnight at 4°C. Plates were washed with PBST (0.1% Tween 20 in PBS) and blocked with 5% FBS in PBST for 1 h at 37°C. Serially diluted mAbs in blocking buffer were added for 2 h at 37°C, followed by HRP-conjugated secondary antibody for 2 h. Then, 3,3',5,5'-tetramethylbenzidine (TMB) substrate (Thermo Fisher Scientific) was added and reaction was stopped with 2M sulfuric acid. OD450nm was measured using a Tecan Infinite M1000 plate reader.

Immunofluorescence assay (IFA)

Cos-7 cells were cultured in Dulbecco's Modified Eagle's Medium (DMEM) (Invitrogen) media on coverslips for 24 h and transfected with the desired plasmids using Xtreme Gene 360 transfection reagent (Sigma-Aldrich). After 24 h, the medium was removed, and the cells were washed twice with phosphate buffered saline (PBS). The cells were fixed with 4% paraformaldehyde for 10 min and permeabilized with 0.2% Triton X-100 for another 10 min. To block non-specific binding, coverslips were incubated with 1% bovine serum albumin for 30 min. The cells were subsequently incubated with mAb 7A7 for 1.5 h. Following washing with PBS, they were incubated with Alexa Fluor 488-conjugated goat anti-mouse IgG secondary antibodies (Invitrogen) for 1 h. After additional washes, the cells were stained with DAPI and mounted for imaging. Images were acquired with Olympus CKX53 microscope using Olympus LCAch N 20x/0.40 iPC objective lens and Olympus DP27 colour camera with Olympus cellSens software.

Virus infection

Vero E6 cells (American Type Culture Collection) were seeded at a density of 40,000 cells per well in a Nunc™ Lab-Tek™ Chamber Slide (Thermo Fisher Scientific) using DMEM supplemented with 10% fetal bovine serum (FBS). After 24 h, the cells were infected with SARS-CoV-2 at a multiplicity of infection of 0.1 in DMEM for 1 h or mock-infected as a control. Following the 1 h infection period, the cells were washed three times with PBS and cultured in DMEM containing 10% FBS. At 48 h post-infection, the medium was removed,

and the cells were washed three times with PBS, then fixed and stained as described above for IFA.

Supplementary Figures

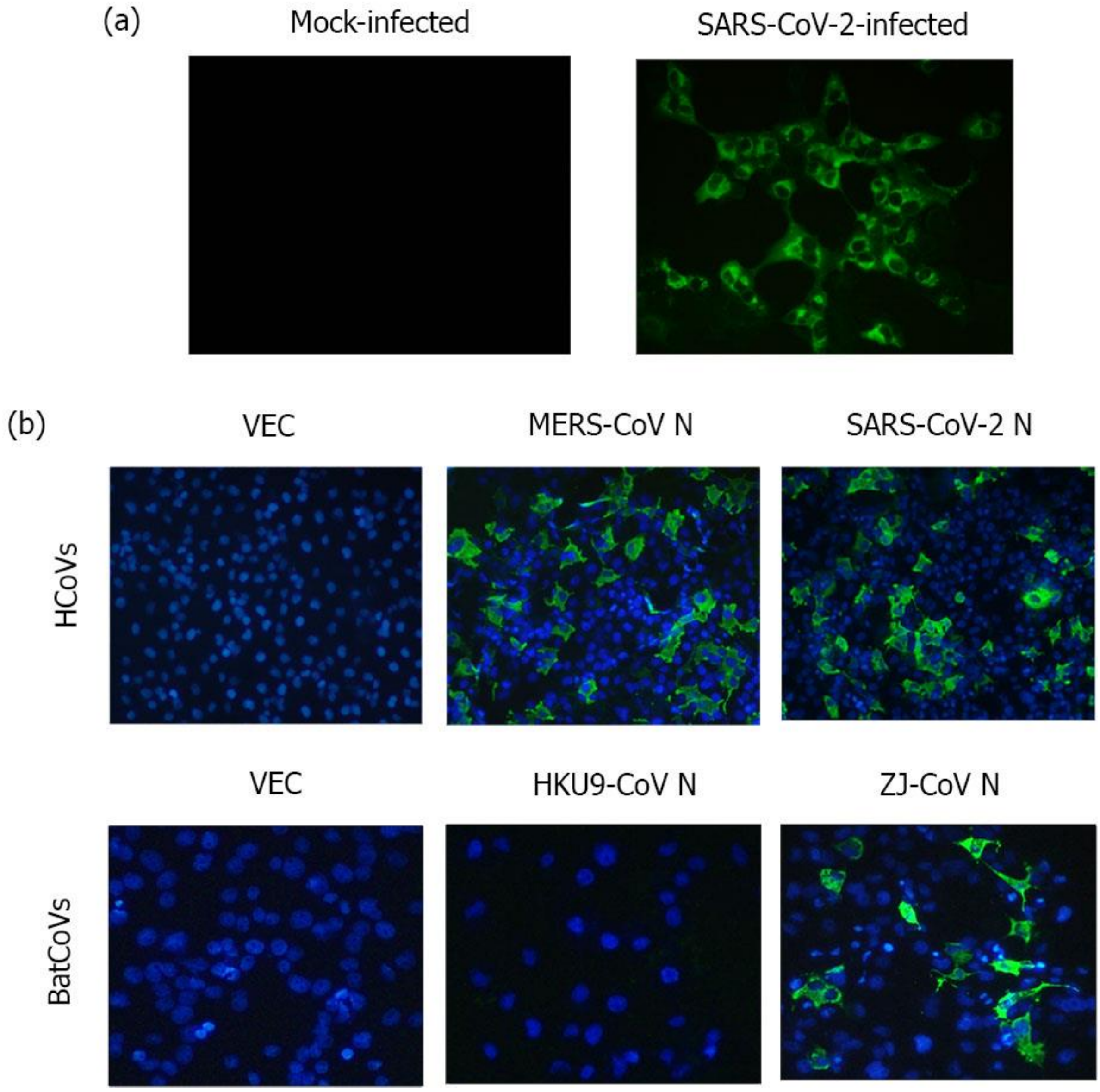

Figure S1. (a) Vero-E6 cells were mock-infected or infected with SARS-CoV-2 (multiplicity of infection of 0.1) and stained with mAb 7A7 at 2 days post-infection. (b) IFA results of Cos-7 cells expressing Flag-tagged vector or N proteins of HCoVs (top panel) or Flag-tagged N protein of BatCoVs (bottom panel) using mAb 7A7, followed by Alexa Fluor 488-conjugated secondary antibody (green) are shown. Cell nuclei were counterstained with DAPI (blue).

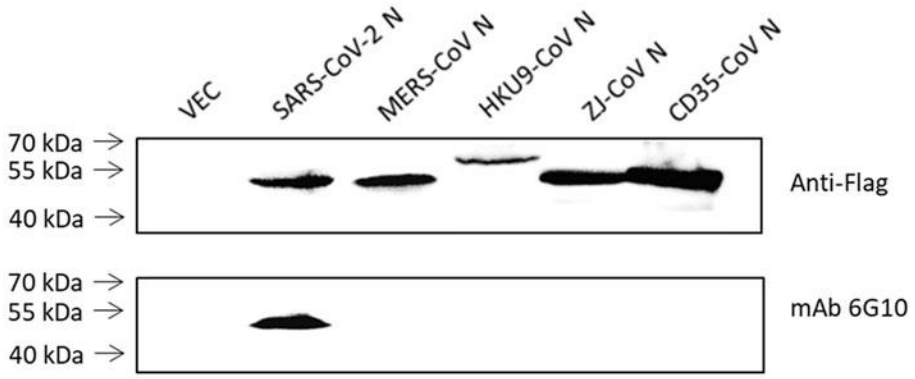

Figure S2. Lysates from 293T cells transiently transfected with Flag-tagged N protein plasmids of 5 different betacoronaviruses belonging to the *Sarbecovirus* (SARS-CoV-2), *Merbecovirus* (MERS-CoV), *Nobecovirus* (HKU9-CoV) and *Hibecovirus* (ZJ-CoV and CD35-CoV) subgenera were obtained and immunoblotted with anti-Flag Ab and mAb 6G10. Empty vector-transfected 293T cell lysate labelled as 'VEC' served as a negative control. The molecular weight (kDa) of the proteins relative to their position on the blots is given on the left side of the panel.

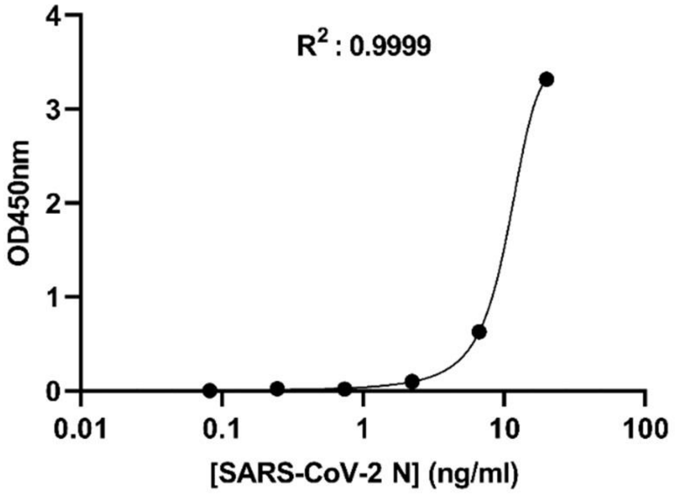

Figure S3. Standard curve between SARS-CoV-2 N protein concentrations and their respective OD_{450nm} from sELISA using 7A7(6G10). R^2 value is calculated based on 4PL regression analysis.

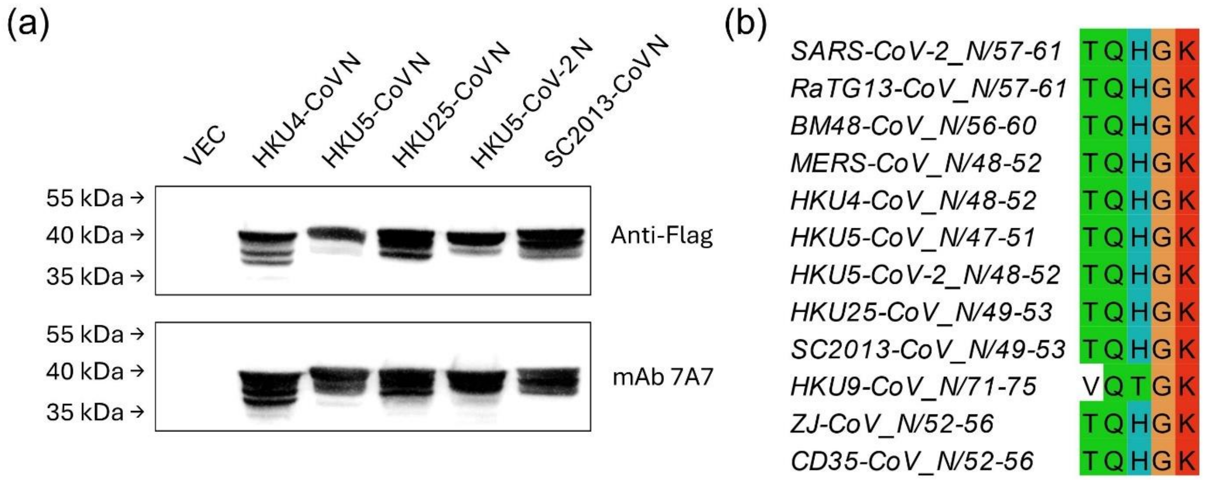

Figure S4. (a) Lysates from 293T cells transiently transfected with Flag-tagged N protein plasmids of 5 different merbecoviruses (HKU4-CoV, HKU5-CoV, HKU25-CoV, HKU5-CoV-2 and SC2013-CoV) were obtained and immunoblotted with anti-Flag Ab and mAb 7A7. The molecular weight (kDa) of the proteins relative to their position on the blots is given on the left side of the panel. (b) Sequence alignment of N proteins highlight the potential region targeted by mAb 7A7.

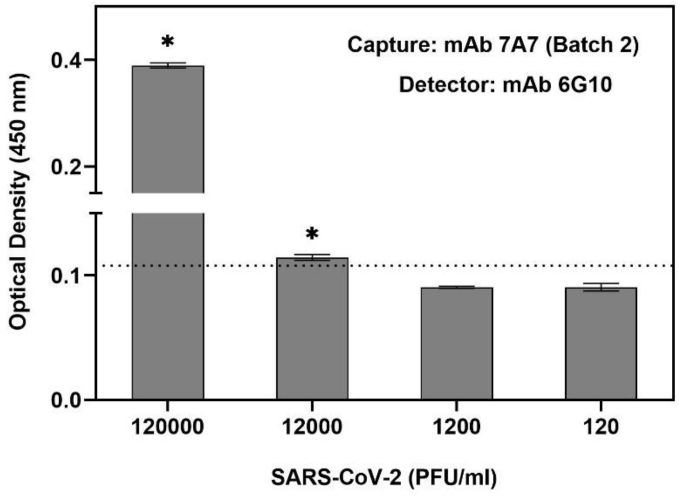

Figure S5. sELISA using a different batch of mAb 7A7 to pair with mAb 6G10 achieves the same LOD for SARS-CoV-2 virions – 12000 PFU/mL (as shown in Fig. 3(b) in the main text). mAb 7A7 and biotinylated-mAb 6G10 were used as capture and detector antibody, respectively, in sELISA of SARS-CoV-2 virions. *$p < 0.05$ represents significant difference compared to blank. Dotted line indicates the cut-off value.

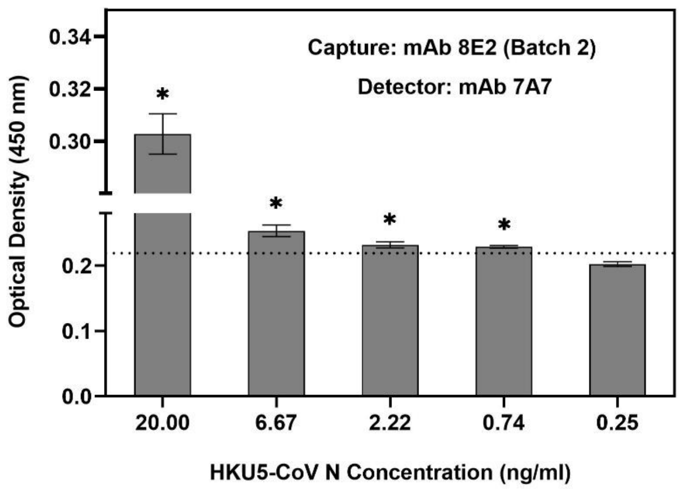

Figure S6. sELISA using a different batch of mAb 8E2 to pair with mAb 7A7 achieves the same LOD for recombinant HKU5-CoV N protein – 0.74 ng/mL (as shown in Fig. 5(b) in the main text). mAb 8E2 and biotinylated-mAb 7A7 were used as capture and detector antibody, respectively, in sELISA of HKU5-CoV N protein. *p < 0.05 represents significant difference compared to blank. Dotted line indicates the cut-off value.

Supplementary Tables

Table S1. Accession numbers of N proteins from different CoVs,

Virus strain	Accession number of N protein
SARS-CoV-2	GenBank YP_009724397.2
MERS-CoV	GenBank YP_009047211.1
ZJ-CoV	GenBank YP_009072446.1
OC43-CoV	GenBank YP_009555245.1
RaTG13-CoV	GenBank QHR63308.1
BM48-CoV	GenBank YP_003858591.1
HKU4-CoV	GenBank YP_001039960.1
HKU5-CoV	GenBank YP_001039969.1
HKU25-CoV	GenBank ASL68949.1
HKU5-CoV-2	GenBase C_AAI84077.1
SC2013-CoV	GenBank AHY61344.1
HKU9-CoV	GenBank ABN10915.1
CD35-CoV	GenBank UUT43655.1

Table S2. Ct values of SARS-CoV-2 virions with titres of 12000 to 120 PFU/ml analysed in qRT-PCR.

Virus (PFU/ml)	Ct value
12000	27.084
1200	30.376
120	33.928

Table S3. N protein levels in the nasal swabs collected from 16 COVID-19 patients determined using sELISA and their Ct values obtained via qRT-PCR.

No.	Patient ID	N protein level (ng/ml)[1]	Ct value
1.	P061	1.40	28.463
2.	P064	9.00	19.100
3.	P067	1.14	27.123
4.	P069	22.08	17.105
5.	P071	2.44	22.075
6.	P072	23.04	17.763
7.	P073	7.44	20.764
8.	P075	1.53	25.073
9.	P088	4.96	21.970
10.	P099	2.33	24.902
11.	P125	1.83	25.010

12.	P133	23.04	17.600
13.	P066	Not detected	28.039
14.	P068	Not detected	33.493
15.	P077	Not detected	28.301
16.	P086	Not detected	26.236

[1] SARS-CoV-2 N protein concentrations in the patient swab samples were determined by interpolating from a standard curve constructed between commercial SARS-CoV-2 N protein concentrations and their respective absorbance in sELISA using 7A7(6G10) pair (Fig. S3).
